# Fellow-Workers and the Roll of Honour

**Published:** 1914-10-10

**Authors:** 


### ROUND THE WORLD.

[The Editor Invites Early Information and Contributions Marked "Fellow-Workers."]

### Fellow-Workers and the Roll of Honour.

Sir James Barr, who holds the rank of lieutenant-colonel in the 1st Western General Hospital (Liverpool), is one of the best known members of the medical profession in this country. An M.D. Glasg. and F.R.C.P. Lond. he is also a Fellow of the Royal Society, and possesses the honorary degrees of LL.D. Toronto and Liverpool. For many years he has taken an interest in things military, and has acted as county director for the West Lancashire Voluntary Aid Association connected with the Territorial Force. Sir James Barr was a popular President of the British Medical Association's Liverpool meeting, and was knighted for his public work in 1905.

Mr. William Alexander, consulting surgeon to the Royal Southern Hospital at Liverpool, and lecturer on clinical surgery in Liverpool University, is also a lieutenant-colonel in the same establishment. He is a well-known North Country consultant. Studying medicine at Queen's College, Belfast, he took the Irish M.D. degree with a gold medal and exhibition in 1870, subsequently proceeding to the F.R.C.S. Eng.

Mr. Rushton Parker, M.B., B.S. Lond., F.R.C.S. Eng., who holds the same rank at Liverpool, entered University College, London, as a student in the early 'sixties, and after qualification pursued his surgical studies in Paris and Vienna. In Liverpool he has had a very distinguished career, and after many years' service at various public institutions there is now consulting surgeon to the Liverpool Royal Infirmary.

The following practitioners mobilised as captains in the 1st Western General Hospital:—Dr. T. R. W. Armour, Dr. H. Armstrong, Dr. R. J. Buchanan, Mr. R. A. Bickersteth, Dr. V. C. de Boinville, Mr. D. Douglas-Crawford, Mr. A. J. Evans, Dr. J. M. Hunt, Mr. C. T. Holland, Dr. Pantland Hick, Dr. J. Hay, Mr. R. E. Kelly, Mr. F. C. Larkin, Mr. J. R. Logan, Dr. K. W. Monsarrat, Dr. L. A. Morgan, Mr. W. Permewan, Mr. J. L. Roberts, and Dr. W. B. Warrington.

The 2nd Western General Hospital has been mobilised at Manchester, where Dr. E. S. Reynolds, Mr. F. A. Southam, and Mr. W. Thorburn are the lieutenant-colonels. The majors of this institution are: Mr. A. H. Burgess, Mr. J. J. Cox, Mr. A. H. Griffith, Sir W. Milligan, Dr. G. R. Murray, Mr. J. H. Ray, and Dr. R. B. Wild.

Dr. Ernest Septimus Reynolds is one of the senior members of the staff at the Manchester Royal Infirmary, where he heads the list of physicians. Qualifying M.R.C.S. Eng., L.S.A., in 1883, he subsequently became an M.D. Lond., and was elected to the Fellowship of the Royal College of Physicians in 1896. Dr. Reynolds is the author of numerous communications on general medicine, and gave special attention to the remarkable neuritis epidemic which occurred in Manchester some fourteen years ago, and in connection with which he made a series of interesting reports to the medical press. As professor of clinical medicine in Manchester University, Dr. Reynolds has been an important influence in medical education at that centre. His past appointments include those of senior physician to the Ancoats Hospital, lecturer on tropical medicine to the Owens College, Manchester, and pathologist to the West Riding Asylum at Wakefield.

Mr. Frederick Armitage Southam has had many years' service and practice in Manchester, where he is now emeritus professor of surgery in the University and consulting surgeon to the Royal Infirmary, as well as to several other institutions. Mr. Southam has been a F.R.C.S. Eng. for nearly forty years, and has taken an active part in the development of modern surgical procedures.

Mr. William Thorburn is another distinguished Manchester surgeon who has attained distinction in the city where, as a student, he first took up medicine. At Owens College Mr. Thorburn had an exceptionally distinguished academic career, in the course of which he secured at least four gold medals. His honours included the gold medal and exhibition in physiology, with honours in chemistry and forensic medicine at the Intermediate M.B. Lond., a gold medal in obstetric medicine in the M.B. Lond. 1884, with the gold medal at the B.S. Lond. in the same year; also the second place in honours in physiology at the B.Sc. Lond., and the Jacksonian prize of the Royal College of Surgeons. Among appointments he holds at present may be mentioned those of senior surgeon to the Manchester Royal Infirmary, professor of clinical surgery in the Manchester University, and examiner in surgery at the London University.

Dr. E. Le C. Lancaster, Mr. O. E. B. Marsh, Dr. H. R. Vachell, and Dr. T. Wallace are the lieutenant-colonels at the 3rd Western General Hospital (Cardiff), where the following practitioners hold the rank of major: Dr. B. W. Broad, Mr. W. F. Brook, Dr. P. R. Griffiths, Dr. A. Howell, Dr. C. Lewis, Dr. D. R. Paterson, Dr. W. M. Stevens.

Dr. Ernest Le Cronier Lancaster is an old student of Oxford University and St. George's Hospital, who holds the post of senior physician to the Swansea Hospital. Formerly he was demonstrator in anatomy and physiology at St. George's Hospital Medical School and resident obstetric assistant in that hospital. He has for many years taken an interest in military medicine, and holds the appointment of civil medical examiner of recruits to the Royal Marines.

Mr. Octavius Edward Bulwer Marsh qualified M.R.C.S. Eng., L.R.C.P. Ed., and L.M. in 1876. For many years he was on the active staff of the Newport and Monmouth County Hospital, where he is now consulting surgeon. He is also a J.P. for the borough of Newport, and is associated with various public institutions there.

Dr. Herbert Redwood Vachell is consulting physician to the King Edward the Seventh Hospital at Cardiff, and honorary examining physician for the Queen Alexandra's Hospital at Davos. A King's College student, he qualified in 1876, and became an M.D. Aberd. in 1881.

Dr. Thomas Wallace qualified as an Irish M.D. forty-four years ago. Settling in Cardiff, he served for many years as surgeon to the infirmary there, eventually becoming a member of the consulting staff. He is a J.P. for Cardiff and Glamorganshire, district surgeon to the St. John Ambulance Brigade, and surgeon to the Bute Docks Police.

